# Emerging manufacturers engagements in the COVID −19 vaccine research, development and supply

**DOI:** 10.1016/j.vaccine.2020.06.022

**Published:** 2020-07-22

**Authors:** Sonia Pagliusi, Stephen Jarrett, Benoit Hayman, Ulrike Kreysa, Sai D. Prasad, Martin Reers, Pham Hong Thai, Ke Wu, Youn Tao Zhang, Yeong Ok Baek, Anand Kumar, Anatoly Evtushenko, Suresh Jadhav, Weining Meng, Do Tuan Dat, Weidan Huang, Samir Desai

**Affiliations:** a DCVMN International, Route de Crassier 7, 1262 Eysins-Nyon, Switzerland; b Gracious International, 28 Jiafeng Road, Shanghai 200131, China; c DCVMN International, Switzerland; d GS1, Avenue Louise 326, 1050 Bruxelles, Belgium; e Bharat Biotech, Hyderabad, India; f Biological E Ltd., Hyderabad, India; g BioNet-Asia, Bangkok, Thailand; h Bravovax, Wuhan, China; i China National Biotech Group, Beijing, China; j EuBiologics, Seoul, South Korea; k Indian Immunologicals Ltd., Hyderabad, India; l St. Petersburg Research Institute of Vaccines and Serums, Russia; m Serum Institute of India, Pune, India; n Sinovac Biotech, Beijing, China; o Vabiotech, Hanoi, Viet Nam; p Innovax Biotech, Xiamen, China; q Zydus Cadila, Ahmedabad, India

## Abstract

The World Health Organization declared the COVID-19 disease as a pandemic requiring a rapid response. Through online search, direct communication with network members and an internal survey, engagements of developing countries’ vaccine manufacturers’ network members in the research and development of COVID-19 vaccines and their capacities in the manufacturing, fill-finish and distribution of vaccines were assessed.

Currently, 19 network members engaged in research and development of COVID-19 vaccines, using six principal technology platforms. In addition, an internal survey showed that the number of vaccines supplied collectively by 37 members, in 2018–19, was about 3.5 billion doses annually. Almost a third of network members having vaccines prequalified by the World Health Organization comply with international regulations and mechanisms to distribute vaccines across borders. The use of existing manufacturing, fill-finish and distribution capabilities can support an efficient roll-out of vaccines against COVID-19, while maintaining supply security of existing vaccines for on-going immunization programmes.

## Highlights

• Broad research and development is key to achieving an effective safe COVID-19 vaccine.
• Currently 19 Network members engaged in research & development of 22 COVID-19 vaccines.
• Collectively 37 Network manufacturers supply around 3.5 billion vaccine doses annually.
• Existing manufacturing capabilities can accelerate the availability of COVID vaccines.
• Deploying available vaccine production capacity, will save time, resources and lives.

## 1 Introduction and background

A new strain of coronavirus not previously identified in humans, the Severe Acute Respiratory Syndrome Coronavirus-2 (SARS-CoV-2), emerged in December 2019 [1]. COVID-19 is the name given to the disease associated with the SARS-CoV-2 virus. Given escalating outbreaks in over 100 countries, the World Health Organization declared on 11 March 2020 that the COVID-19 disease can be characterized as a pandemic [2]. Early June 2020, the total number of cases identified passed 7.8 million with over 430 thousand deaths reported globally [3].

Coronaviruses are enveloped positive stranded RNA viruses in the order of Nidovirales [4]. Epithelial cells in the respiratory and gastrointestinal tract are the primary target cells of these viruses. COVID-19 is highly contagious which would indicate the need for widespread vaccination, once vaccines are available. The genetic sequence of the novel coronavirus was shared for developing specific diagnostic and other health products including vaccines [5].

The Developing Countries Vaccine Manufacturers Network (DCVMN) is a public-health driven alliance representing vaccine manufacturers from emerging countries engaged in research, development, manufacturing and supply of vaccines for local and international use, aiming to protect all people against known and emerging infectious diseases [6]. DCVMN members have proven manufacturing, formulation, filling, packaging and distribution capabilities to ensure vaccines reach populations round the world in support of the global call to fight the pandemic [7]. This report outlines four key areas where DCVMN-affiliated manufacturers are engaged in vaccine research and development using various technology platforms, and could play a role in large scale manufacturing and supply capabilities, for supporting an efficient roll-out of potential vaccines against COVID-19.

## 2 Methodology

We conducted an online search, on COVID-19 vaccine research and development activity by DCVMN members, to compile an overview of initial efforts to date, combined with recent survey results on supply capacity as described in detail elsewhere [6],complemented with some data from 2019. To enable unconflicted responses, individual data regarding number of doses supplied were agreed to remain confidential. Data collected include developments reported through the WHO’s Blueprint updated list [8], information identified from the internet and publicly available sources, reports of scientific meetings and direct communication with DCVMN members. The information is shared in a summarized manner with global health stakeholders to help improve coordination in the COVID-19 response and to achieve the leverage and optimal use of resources and capabilities .

## 3 Research and Development Efforts and Vaccine Technology Platforms

Of over 125 candidate vaccines from various manufacturers and academic institutions worldwide [8], [9], 19 DCVMN members have rapidly engaged in the research and development of 22 COVID-19 candidate vaccines (Table 1). Four members have candidate vaccines in Phase 1 or 1/2 trials and the others are in the preclinical stage, as of the end of May 2020. Eight of the 19 members’ manufacturers developing COVID vaccines have vaccines prequalified by WHO.

**Table 1:** List of DCVMN Member companies currently engaged in COVID-19 vaccine research and development. The data reported on the table include information on manufacturer name and location (first column); WHO Prequalification status of other available vaccines; technology platforms used for COVID-19 vaccine development (second column); the current phase of vaccine development (third column); compliance to global traceability standards for international supply, as provided by GS1, to satisfy GAVI/UNICEF requirements ​[16]​; technical information publicly available; source of information. (*) www.gs1.org ; (**) Biomanguihos and Butantan are DCVMN members, and collaborate to develop one candidate vaccine. Sources from information: (1) https://extranet.who.int/gavi/PQ_Web/; (2) https://www.who.int/who-documents-detail/draft-landscape-of-covid-19-candidate-vaccines; (3) https://www.news18.com/news/india/human-trials-of-bharat-biotechs-covid-19-nasal-drop-vaccine-to-begin-in-4-months-2568711.html; (4) Communication from Eubiologics, 6 May 2020; (5) https://economictimes.indiatimes.com/industry/healthcare/biotech/pharmaceuticals/serum-institute-to-be-ready-with-coronavirus-vaccine-by-2022/articleshow/74212495.cms?utm_source=contentofinterest&utm_medium=text&utm_campaign=cppst; (6) https://www.pharmaceutical-business-review.com/news/sinovac-biotech-covid-19-vaccine-trial/; (7) https://www.biospectrumindia.com/news/43/15795/zydus-to-develop-vaccine-against-covid-19.html; (8) http://www.bionet-asia.com/media/news-events/; (9) https://www.geovax.com/news/geovax-and-bravovax-wuhan-china-to-collaborate-on-development-of-coronavirus-vaccine; (10) https://www.indimmune.com/mediia/iil-news; (11) https://www.trialsitenews.com/gsk-joins-xiamen-innovax-biotech-to-support-covid-19-vaccine-development-expands-activity-to-fight-war-against-pandemic/; (12) https://russkiymir.ru/en/news/271406/; (13) https://e.vnexpress.net/news/news/vietnam-tests-covid-19-vaccine-on-mice-4093317.html.

DCVMN Member and location	Vaccine technology platform	COVID-19 vaccine development status	Manufacturer using GS1* traceability standards	Technical information (publicly source available)
**Manufacturer with WHO prequalified vaccines**				1
Beijing Institute of Biological Products, China National Biotec Group, China	Inactivated virus	Phase 1/2		Beijing Institute of Biological Products is developing an inactivated vaccine which is in a Phase 1/2 trial. (2)
Bharat, India	Non-replicating viral vector	Pre-clinical	YES	Bharat Biotech in collaboration with the University of Wisconsin–Madison and the vaccine company FluGen has begun the development and testing of a vaccine to be delivered intranasally. It will be based on an influenza virus where gene sequences from SARS-CoV-2 are inserted into M2SR- vaccine platform (M2-ion channel protein Deficient Single Replication) which has completed Phase II trials in the USA. Bharat will manufacture clinical lots of the vaccine. (3)
Biological E, India	Recombinat Protein sub-unit	Pre-clinical	YES	Biological E is developing an adjuvanted subunit vaccine comprising the Receptor or Binding Domain (RBD) of SARS-COV-2 spike protein as the antigen candidate. (2).
Eubiologics, Republic of Korea	Recombinat Protein sub-unit	Pre-clinical	YES	EuBiologics is in a vaccine in consortium with Korean companies and the BSL-3 research institute. It is developing a protein sub-unit vaccine with platform technology of critical antigen and adjuvant formulation technology with TLR4 agonist. EuBiologics is conducting neutralizing antibody test in mice and in vivo proof of principle in Ferret, with adjuvant formulation of each key antigen. (4)
Biomanguinhos/Fundaçāo Oswaldo Cruz and Instituto Buntantan, Brazil**	Replicating viral vector	Pre-clinical		Fundaçāo Oswaldo Cruz and Instituto Buntantan are developing an attenuated influenza expressing an antigenic portion of the Spike protein. (2)
Serum Institute of India, India	Live attenuated virus (2 candidates)	Pre-clinical	YES	The Serum Institute of India Pvt. Ltd. (SIIPL) is developing a live attenuated vaccine, in partnership with Codagenix and a measles based viral vectored vaccine, with Themis, both in pre-clinical stage. (5) In addition, SIIPL is also working on VPM1002 as immune enhancer and has started Phase-3 in India. Trials were also initiated in Germany [11] and will start shortly in Canada, Australia and New Zealand.
Sinovac, China	Inactivated virus (2 candidates)	Pre-clinical, Phase 1/2		Sinovac Biotech, in Beijing, is developing an inactivated vaccine adjuvanted with alum currently in Phase 1/2, and another inactivated vaccine with Dynavax currently in pre-clinical. (6)
Zydus Cadila, India	DNA; Replicating viral vector (2 candidates)	Pre-clinical	YES	Zydus Cadila in India is developing two candidate vaccines. The first deals with a DNA vaccine against the major viral membrane protein responsible for the cell entry of the novel coronavirus, expected to enter the preclinical toxicology studies in Q2/2020. Thereafter, Zydus will undertake the clinical development and licensure of this vaccine. This candidate vaccine can be manufactured in biosafety level 1, and be prepared in many facilities to make billions of doses for global use. It can also address antigenic drift or shift of the virus, developing a modified construct in 2-3 weeks. The second approach deals with a live attenuated recombinant measles virus vectored vaccine. (7)
**Manufacturer without WHO prequalified vaccines**				
Minhai Biotechnology, China	Inactivated virus	Pre-clinical		Beijing Minhai Biotechnology is developing an inactivated vaccine, currently in pre-clinical stage. (2)
BioNet-Asia, Thailand	DNA	Pre-clinical		BioNet-Asia in Thailand is developing a COVID-19 GENE-based vaccine (COVIGEN) encoding the S (Spike) protein of SARS-CoV-2, and is actively collaborating with different organizations in Thailand and internationally. BioNet’s DNA based vaccine candidate is currently undergoing pre-clinical testing using various delivery systems, and a phase 1/2 trial is planned for Q3 2020. (8)
Bravovax, China	Non-replicating viral vector (2 candidates)	Pre-clinical		BravoVax, in Wuhan, China, and GeoVax Labs Inc. have signed a Letter of Intent to jointly develop a vaccine. GeoVax’s Modified Vaccinia Ankara (MVA) platform technology, which elicits protective T cell as well as antibody responses, can be combined with the potent immunogenicity of Virus-Like Particles (VLPs). BravoVax will provide testing and manufacturing support, as well as interactions with public health and regulatory authorities. BravoVax also has an in-house adeno-vectored COVID-19 candidate vaccine entering preclinical studies. (9)
Indian Immunologicals, India	Live attenuated virus	Pre-clinical	YES	Indian Immunologicals Ltd. has entered into a collaboration agreement with Australia’s Griffith University to develop a live attenuated vaccine using the latest codon de-optimization technology, and its existing Vero cell platform technology for mass production of the candidate vaccine. (10)
Innovax, China	Recombinat Protein sub-unit	Pre-clinical		Innovax from Xiamen, China, in collaboration with GSK and Xiamen University, is developing a recombinant candidate vaccine based on COVID-19 recombinant truncated S (Spike) proteins (XWG-03) currently in pre-clinical stage. (11)
Institute of Medical Biology, Chinese Academy of Medical Sciences, China	Inactivated virus	Phase 1		The Institute of Medical Biology is developing an inactivated vaccine currently in Phase I. (2)
Medigen Vaccine Biologics Corporation, Taiwan	Recombinat Protein sub-unit	Pre-clinical		Medigen is developing a protein sub-unit vaccine based on spike protein (S-2P protein) + CpG1018 (2)
St. Petersburg Research Institute (SpbNIIVS), Russia	Recombinat Protein sub-unit	Pre-clinical		The Saint-Petersburg Scientific Research Institute of Vaccines and Serums (SpbSRIVS), in Russia, is developing recombinant protein nanoparticles, based on S (spike) protein and other epitopes. (12)
Vabiotech, Vietnam	Recombinat Protein sub-unit	Pre-clinical		Vabiotech in Vietnam, has started developing a vaccine based on the baculovirus expression system with the University of Bristol, UK, and Imperial College, London, within activities of the Future Vaccine Manufacturing Research (FVMR) Hub. (13)
Wuhan Institute Biological Products, China National Biotec Group, China	Inactivated virus	Phase 1/2		Wuhan Institute of Biological Products, under the China National Biotech Group, is developing an inactivated vaccine, entering Phase 2 trial for age-groups 6 years and above. (2)

Among the developments, six principal platforms are being pursued worldwide [10]:

1. Live attenuated virus
2. Inactivated virus
3. Nucleic acid: DNA and RNA^1^
4. Replicating viral vectors (e.g. measles)
5. Non-replicating viral vector (e.g. adenoviral vectors and Modified Vaccinia Ankara, MVA)
6. Recombinant protein sub-unit or Virus-Like Particles.

The feasibility of any of these platforms to use existing manufacturing capacity has to be assessed. Formulation combined with adjuvants may potentially play a role in the immunogenicity and efficacy of candidate vaccines. Interestingly, it has been suggested that immune modulation by Bacille Calmette–Guérin (BCG) could provide protection against COVID-19 [11] and this hypothesis is being tested in randomized clinical trials by at least one DCVMN member. The efficacy of the Recombinant BCG vaccine VPM1002 is in phase 3 study as part of COVID-protection [12].

It is not yet determined what the degree of natural immunity infection elicits, nor what the level of herd immunity, if attainable, might protect people against the viral infection. It is assumed at this stage that vaccination of all populations will be demanded, given the high level of contagion of the virus. It is conjectured that a number of vaccines will be necessary to supply the world’s populations. As yet, there is no determination as to whether any candidate vaccine will be universal or indicated for specific populations, how many doses will be required, nor of the likely container presentations: prefilled single dose syringes, single or multidose vials, nasal spray, micropatches or other delivery devices.

## 4 Rapid scale up and large scale manufacturing

While broad research and development is key to achieving an effective safe vaccine, massive levels of manufacturing will be required to meet the presumed high demand. Speed of transitioning from a proven vaccine to large-scale manufacturing will be essential in order to commence immunization globally as soon as possible. Deploying available facilities, especially viral vaccine production capacity, will likely save time, resources and, importantly, lives, recognizing DCVMN members' capacities, both those with WHO prequalified vaccines and those certified by national regulatory authorities. [6].

According to an internal survey the total number of doses supplied collectively by 37 DCVMN members was around 3.5 billion in 2018/2019 (Table 2), ranging from 50 thousand to 1.5 billion doses per member company. This amount corresponds in most cases to filling capacity for single dose syringes, single or multidose vials, under routine operations, notably one shift Monday-Friday. If operations were adjusted to run additional shifts, the capacities could be increased. The survey assessed supplied doses in 2018–19 only; number of vaccine doses produced, e.g. bulk in stock, or batches not released, or in storage, were not assessed. Noteworthy, some of the vaccines supplied included monovalent vaccines such as tetanus, hepatitis B (HepB), Japanese encephalitis, BCG, rabies, rubella, Haemophilus influenzae type b (Hib), Meningitis A, monovalent Poliovirus, typhoid, varicella, Enterovirus-71, and Hepatitis E. Other supplied vaccines included combination vaccines based on two, three, four or five antigenic components, such as Diphtheria-Tetanus-Pertussis (DTP), Measles-Rubella or Measles-Mumps-Rubella, bivalent-trivalent- and quadrivalent meningitis vaccines, five-valent rotavirus vaccines, tri- and quadrivalent seasonal influenza vaccines, bivalent Human Papillomavirus, ten- or thirteen-valent Pneumococcal conjugate, bivalent cholera vaccine, bivalent poliovirus and trivalent inactivated poliovirus (IPV) vaccines, pentavalent vaccines (DTPHepBHib) as well as a hexavalent (DTPHepBHib + IPV). Thus, it is suggested that the collective antigen manufacturing capacity of this group of manufacturers is higher than 3.5 billion doses annually. In addition, 4 companies did not respond to the survey, and may have additional available capabilities. Assuming that collectively manufacturers on average were able to dedicate 50% of their existing capacities to vaccines against COVID-19, manufacturing and filling billions of doses could be attained in the short term, enabling global access to these new vaccines. Filling a potential COVID-19 vaccine into ten dose vials, as used in large immunization programmes, would imply that with filling 100 million vials, one billion doses could be supplied.

**Table 2:** List of the broad range of vaccine supply capabilities from 37 DCVMN member manufacturers, in 2018–19. First column shows the various levels of supply capacity from different manufacturers. Second column shows the number of respondents that reported the number of doses supplied in 2018–19. Third column denotes the number of manufacturers with WHO prequalified vaccines within each level of supply. Fourth column shows the number of vaccine doses collectively supplied in 2018–19 by manufacturers within the same level of capacity. Total number of vaccine doses collectively supplied in 2018–19, self-reported by 37 respondents ,was 3′456′079′910 doses. Number of doses include all vaccines, if monovalent or multivalent, and also all presentations, if single or multidose vials. Four (4) member manufacturers did not respond to the survey on number of doses supplied.

Level of capacity in supply of vaccines in 2018/19 (in million doses)	Number of DCVMN members that reported vaccines supplied in 2018/19	Number of DCVMN members with WHO PQed vaccines per capacity level	Number of doses collectively supplied by members per capacity level in 2018/19
0	4	0	0
Up to 1	4	1	1.053.000
1–10	7	1	36.966.910
10–20	5	2	81.020.000
20–50	8	2	217.960.000
50–100	3	2	231.300.000
100–200	2	1	267.070.000
More than 200	4	4	2.621.710.000
**Total**	37*	**13**	**3.456.079.910**

* 4 member companies did not respond to the survey.

However, the exact available capabilities, for both antigen manufacturing and filling capacity, and the feasibility and timing of a manufacturing “switch” from currently produced antigens to produce potential COVID-19 vaccines, need to be carefully assessed in discussions with manufacturers on an individual basis.

Gavi, the Vaccine Alliance, estimated that the cost of manufacturing hundreds of millions of doses of a single product will range from $50 million dollars for companies with existing facilities and trained personnel, to about $700 million dollars for those starting from scratch [13].

## 5 Fill-finish and distribution capabilities

To date, DCVMN represents 41 manufacturing member companies across Latin America, Africa, the Middle East and Asia. These companies have established, validated and GMP certified facilities, already providing vaccines to 170 countries. Of these, 13 members have WHO-prequalified vaccines and are familiar with international supply regulations and mechanisms, including international standards, packaging requirements, labelling and regulatory pathways to distribute vaccines safely across borders. Deploying available facilities will likely save time, resources and, importantly, lives, recognizing DCVMN members capabilities, both those with WHO prequalified vaccines and those certified by national regulatory authorities.

The process of filling and finishing vaccines, the last stage in vaccine manufacturing, is where bottlenecks are most likely to occur [13]. Excess capacity is likely to be available at influenza vaccine facilities dedicated to only one hemisphere vaccine. For others, the approach of adding shifts may be able to help to exploit capacity within existing facilities, if two shifts can be managed. Filling capacity for COVID vaccines is being built now, particularly in manufacturers that have a large number of staff and a large footprint.

Notably, 14 member manufacturers with available spare capacity for formulation, fill-finish, labelling, packaging, storage and distribution have voluntarily and publicly declared their technical capabilities [14]. These manufacturers are open to take up the challenges of surge manufacturing or filling, particularly once a potential vaccine against COVID-19 becomes available.

Furthermore, the worldwide unique identification of vaccines will play an important role in creating an efficient supply chain, ensuring vaccines reach those who need them and helping to uniformly document the vaccination of individuals. Often the supply chain is broken, vaccines are expired or not stored correctly, inventory management is not optimal and traceability is not achievable, thus responsibility towards procurers not fulfilled and vaccines are not reaching the populations. In assessing the programmatic suitability of vaccines for WHO prequalification, the Vaccine Presentation and Packaging Advisory Group (VPPAG) recommended improvements by using GS1 barcodes on all vaccine packaging levels used by manufacturers, with the exception of primary packaging [15]. GS1 is a not-for-profit organization^2^ that develops and maintains global standards for business communication, including the barcode, printed on products that allows automatic data capture. The GS1 standards and associated specifications are used to encode the Global Trade Item Number (GTIN), lot number and expiration date, and were recommended for the global vaccine supply chain [16]. Many DCVMN members already comply with the Gavi/UNICEF requirements of adhering to GS1 barcoding traceability standards for international shipping/supply [16]. It will also help to secure the supply chain against substandard and falsified vaccines and provide visibility for all supply chain actors.

## 6 Maintaining supply of other essential vaccines

An important concern is that efforts to make vaccines to halt this pandemic should not hamper other vital vaccine production. DCVMN members are engaged in the production or distribution of nearly 50 distinct vaccines with close to 200 products [6]. Ensuring the security of this supply to national and international destinations is central to the mission of DCVMN. The amount of spare capacity available at any time for the formulation or fill-finish of COVID-19 vaccines will depend on the specific situation of each member which is retained by them as confidential information.

Using spare capacity and/or expanding working operations, DCVMN members can support the manufacturing, fill-finish and distribution of COVID-19 vaccines, without jeopardizing the ongoing production of vaccines for national immunization programmes.

## 7 Conclusion

DCVMN is collaborating with global health authorities, international organizations and vaccine developers to support the development of vaccines against COVID-19. Not only are several of its members involved in the research and development of vaccines but many have formulation and fill-finish capacities available. In this period of the COVID-19 pandemic, the DCVMN constituency also calls for the attention of governments to make every effort to ensure the routine immunization of children and adults through efficient and timely distribution of available vaccines within the respective countries.

It is to be noted that there are other emerging vaccine manufacturers, with WHO-prequalified vaccines, that are not DCVMN members. Furthermore, there are other emerging vaccine manufacturers not captured in this report, as they are not DCVMN members nor have WHO-prequalified vaccines.

Thus, it is possible to anticipate that the world has the capacity to rapidly manufacture, fill-finish, and supply needed COVID-19 vaccines. Nevertheless, details about the capacity for quality control, supply-chain and delivery capabilities need to be closely assessed. DCVMN serves as a platform to keep a communication channel between member manufacturers and the global immunization community, sharing information in an open and equitable manner, galvanizing international cooperation, enhancing country immunization systems and enabling opportunities for all to understand and contribute to advancing the potential for reaching solutions to the COVID-19 pandemic.

## Declaration of Competing Interest

The authors declare that they have no known competing financial interests or personal relationships that could have appeared to influence the work reported in this paper.

## Footnotes

2 www.gs1.org

## Footnotes

1 DNA=Deoxyribonucleic acid ; RNA= Ribonucleic acid
